# POM121 is identified as a novel prognostic marker of oral squamous cell carcinoma

**DOI:** 10.7150/jca.33368

**Published:** 2019-07-24

**Authors:** Haoran Ma, Lijuan Li, Lizhou Jia, Aixiu Gong, Aitao Wang, Lingli Zhang, Mingyan Gu, Genxiong Tang

**Affiliations:** 1 Department of Stomatology, Children's Hospital of Nanjing Medical University, Nanjing, China; 2 Key Laboratory of Antibody Technique of National Health and Family Planning Commission, Nanjing Medical University, Nanjing, China; 3 Department of Anesthesiology, Inner Mongolia Autonomous Region People's Hospital, Hohhot, China; 4 Department of Ophthalmology, Inner Mongolia Autonomous Region People's Hospital, Hohhot, China; 5 Department of Stomatology, Nanjing Maternity and Child Health Care Hospital, Nanjing, China

**Keywords:** POM121, oral squamous cell carcinoma, prognosis, tissue microarrays

## Abstract

**Background:** The aim of this study was to confirm the role of nuclear pore membrane protein 121(POM121) in oral squamous cell carcinoma and to explore the underlying mechanism.

**Methods:** POM121mRNA and protein expressions were evaluated in OSCC tissues and normal oral tissues by quantitative real-time polymerase chain reaction (qRT-PCR) and immunohistochemistry. The relationship between POM121 expression and clinical characteristics was analyzed. Bioinformatics analysis was performed to explore the possible mechanisms how POM121 affected OSCC.

**Results:** We confirmed that POM121 mRNA expression in OSCC tissues was significantly higher than that in non-tumorous tissues, as was POM121 protein expression. POM121 expression was associated with distant metastasis and TNM stage. Multivariate analysis confirmed POM121 expression as an independent prognostic factor for OSCC patients. OSCC patients with high POM121 expression had a worse overall survival (OS) compared with patients with low POM121 expression. Bioinformatics analysis indicated POM121 may regulate OSCC through hedgehog and /or p53 signaling pathway.

**Conclusion:** Targeting of POM121 expression levels could provide new diagnostic and therapeutic strategies for OSCC patients.

## Introduction

Oral cavity cancer is a prevalent malignancy of the head and neck, and an estimated 300,400 new cases and 145,400 deaths occurred globally in 2012^1^. Oral squamous cell carcinoma (OSCC) accounts for 90% of oral malignant cancers, with the 5-year survival rate below 60% 2-4. In addition to smoking, alcohol and HPV infection, betel quid is also a major risk factor for OSCC in southeast Asia 5-7. Due to its special anatomical structure, abundant blood vessels and lymphatic vessels, metastasis to cervical lymph nodes or distant organs is the primary cause of mortality in OSCC patients 8, 9. Therefore, the identification of novel biomarkers and the exploration of underlying mechanisms for metastasis are necessary to improve the clinical management of OSCC patients.

Nuclear pore complexes (NPCs) are large protein cylinders composed of more than 30 nucleoporins (NUPs) that function as the only gateway in the nuclear envelope. NPCs control the communication between the cytosol and the nucleus, and regulate cellular signaling. It was reported that NPCs are involved in cell cycle regulation 10, heterochromatin reorganization^11^, transcriptional regulation^12^, and RNA processing^13^. Aberrant nucleocytoplasmic transport and the subsequent nucleoporin-associated mislocalization of proteins are responsible for tumorigenesis and proliferation 14. NUP98, TPR and several other NUPs have been confirmed to have a close relationship with tumors 15-17, but the specific NUPs and underlying mechanisms connected with OSCC require further investigation.

In this study, we analyzed POM121 expression in OSCC tissues and in oral non-tumor tissues and confirmed that POM121 expression was related to tumor proliferation, metastasis, and poor prognosis in OSCC. Our results provide a new insight into the biological characteristics of POM121 in OSCC. Further studies are required to confirm POM121 as a potential biochemical marker for OSCC patients.

## Materials and methods

### Tissue specimens and patient data

Oral tissue was obtained from 298 patients who had undergone surgery or biopsy at the Affiliated Hospital of Nantong University between January 2003 and December 2012. The tissues consisted of 298 OSCC, 50 dysplasia, and 32 normal oral tissue. None of the patients had undergone any neoadjuvant therapy before sample collection. The patients included 134 males and 164 females, with a median age of 35 years (range, 40-75years). Clinical data was collected from patient medical records, and disease clinical stages of OSCC were determined according to the 7th AJCC Cancer Staging Manual. The study complied with the requirements of the Human Ethics Committee of Affiliated Hospital of Stomatology, Nanjing Medical University, and written consent was obtained from all patients.

### Quantitative real-time polymerase chain reaction (qRT-PCR)

Total RNA in frozen tissue was extracted using Trizol reagent (Invitrogen, Carlsbad, USA) according to the manufacturer's instructions. cDNAs were synthesized using a Maxima First Strand cDNA Synthesis Kit (Invitrogen, Carlsbad, USA) according to the manufacturer's protocol. Amplified reactions were performed with SYBR Select Master Mix (Applied Biosystems, USA) using an ABI Prism 7500HT Sequence Detection System (Applied Biosystems, USA). Primers were as follows: human *POM121* forward, 5ʹ-CAGAGCACACCGTTTGCCT-3ʹ, and reverse, 5ʹ-GATCCCGCACCAATGGAAAAT-3ʹ, and GAPDH forward, 5ʹ-ACAACTTTGGTATCGTGGAAGG-3ʹ, and reverse, 5ʹ-GCCATCACGCCACAGTTTC-3ʹ. POM121 mRNA expression was normalized to GAPDH and calculated as2^-ΔΔCt^ (ΔCt = Ct_POM121_ -Ct_GAPDH_). All experiments were performed three times.

### Construction of tissue microarrays (TMA) and immunohistochemistry analysis (IHC)

TMAs were constructed in the Department of Pathology, Affiliated Hospital of Stomatology, Nanjing Medical University. Tissue cores (2 mm in diameter) were collected from donors and arranged in paraffin that was then made into new FFPE blocks. Sections of 4μm-thick were cut and attached to glass microscope slides. After deparaffinization and rehydration, tissues were heated in 0.01 M citrate buffer, pH 6.0 for antigen retrieval, and then incubated in 3% H_2_O_2_ to inhibit endogenous peroxidase activity 18. POM121 was detected with a polyclonal rabbit anti-human POM121 antibody (dilution 1:200; GTX102128, GeneTex, USA) and EnVision peroxidase kit (Dako, Carpinteria, USA). IHC results were evaluated by two pathologists blind to patient data. Staining intensity for POM121 was scored as 0 (negative), 1 (weakly positive), 2 (moderately positive), and 3 (strongly positive) 19. The final staining score = 3×percentage of strong staining + 2×percentage of moderate staining+ 1×percentageof weak staining. The final staining score ranged from 0 (no staining) to 300 (100% of cells with strongly positive staining). The cutoff point of high and low or no POM121 expression was set as 110.

### Gene set enrichment analysis (GSEA) and protein interaction network

The Cancer Genome Atlas (TCGA) database was searched to obtain RNAseq data of gastric cancer patients. GSEA v2.2.2 software was utilized to perform GSEA and analyze differences in biologic pathway. POM121 mRNA expression level was divided as low (≤0.5) and high (>0.5) groups, and functional gene sets were defined according to KEGG gene sets in Molecular Signatures Database (MSigDB). p < 0.05 and FDR < 0.01 were recognized as differentially expressed threshold. STRING v10.5 was used to construct the putative protein interaction network of POM121.

### Statistical analysis

All data were analyzed using the SPSS 18.0 statistical software package (SPSS Inc, USA). Unpaired Student's t-test was performed to compare two groups. The association between POM121 and clinicopathological features was calculated by Pearson χ2 test. The Kaplan-Meier method was used to analyze cumulative survival rate. The Cox proportional hazard regression model was constructed for univariate and multivariate analyses to confirm the prognostic factors. *P* values of < 0.05 were defined as statistically significant.

## Results

### POM121 mRNA was overexpressed in OSCC tissues

**Figure 1:**
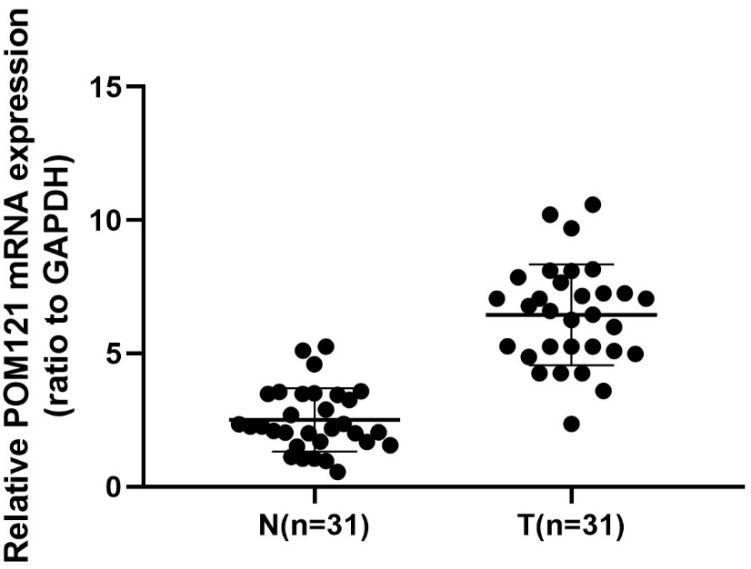
** POM121 mRNA expression in 31 OSCC tissue pairs.** POM121 mRNA expression was examined by qRT-PCR and normalized to GAPDH. POM121 mRNA levels were higher in the 31 OSCC tissues (T) than that in matched normal tissues (N) (*P*< 0.05).

qRT-PCR was performed to detect POM121 mRNA expression levels in 22 cases of OSCC tissues and matched normal oral tissues. Results showed that POM121 mRNA expression in OSCC tissues was 3.312±2.143 fold higher than that in matched normal oral tissues (*P*<0.05, Figure 1).

### POM121 protein expression in OSCC tissues

**Figure 2:**
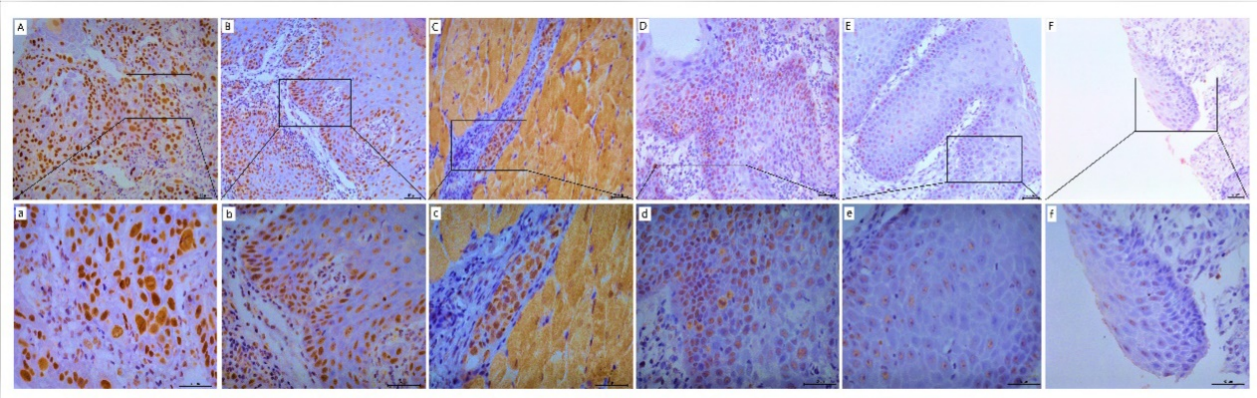
** Representative images of POM121 protein expression in oral tissues. (A)** Poorly differentiated OSCC with high POM121 expression. **(B)** Well differentiated OSCC with high POM121 expression. **(C)** Vascular cancer embolus with high POM121 expression.** (D)** Severe dysplasia.** (E)** Mild dysplasia.** (F)** Normal oral mucosa epithelium. (Original views×20, enlarged views×40)

POM121 protein expression was detected by IHC assay in OSCC, dysplasia, and normal oral tissues. Results showed that POM121, which is embedded in the nucleus and nuclear envelope, was overexpressed in OSCC tissues (Figure 2). The occurrence rate of high POM121 expression in OSCC tissues was 59.73% (178/298), higher than that in normal oral tissues (18.75%, 6/32) and dysplasia (22.0%, 11/50) (Table 1).

### Correlation between POM121 expression and clinical characteristics in OSCC

The correlation between POM121 expression and pathological parameters in OSCC was analyzed. High expression of POM121 was significantly connected with distant metastases (χ^2^=7.680, *P*=0.006) and TNM stage (χ^2^= 5.352, *P*=0.021). However, no significant difference was found between POM121 expression and sex, age, location, differentiation, tumor size, lymph node metastases, caries, or smoking (Table 2).

### Association between POM121 expression and prognosis in OSCC patients

**Figure 3:**
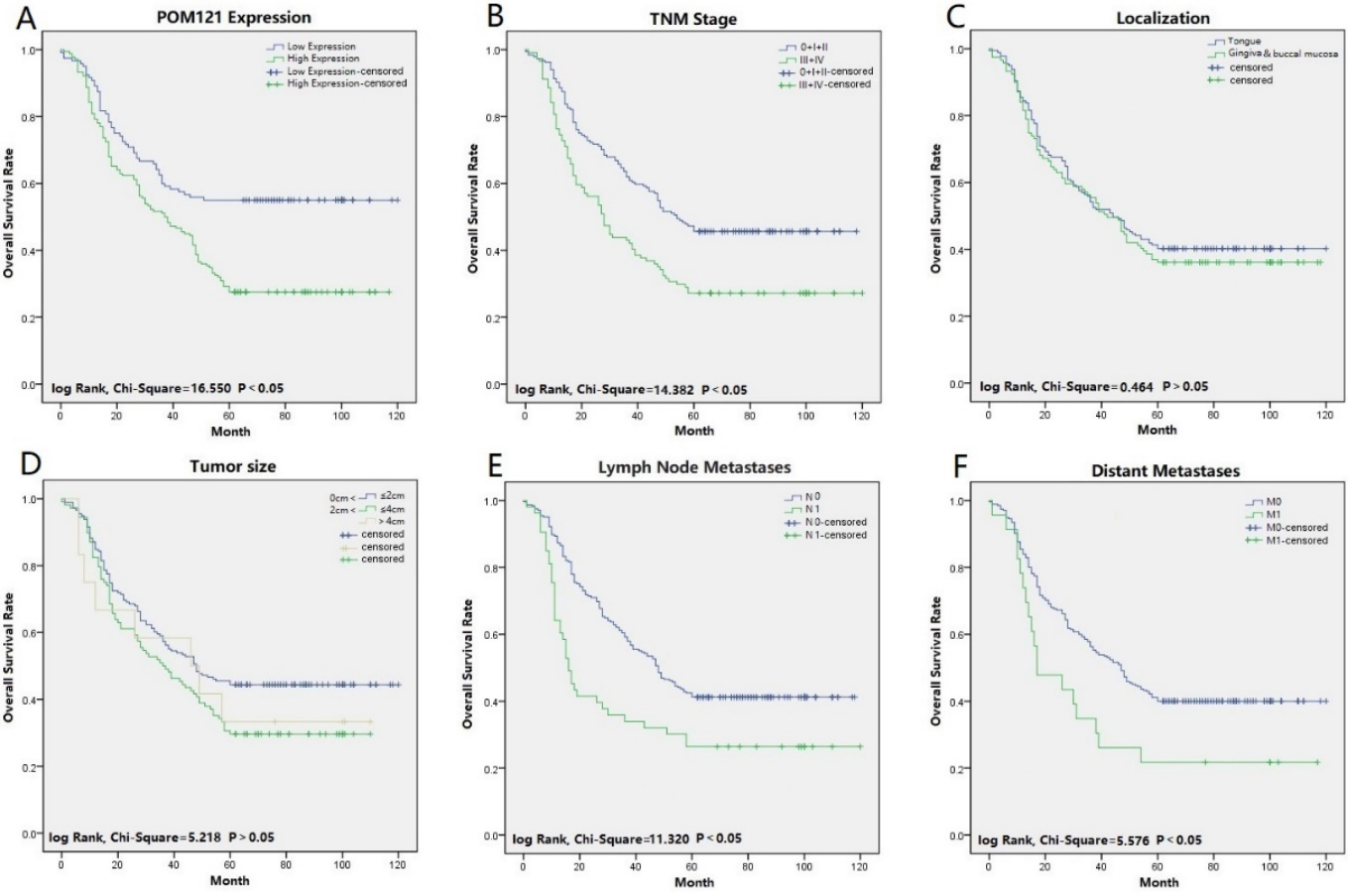
**Survival curves of OSCC using the Kaplan-Meier method and the log-rank test. (A)** Overall survival curves of POM121 (blue line low, green line high).**(B)** Overall survival curves of TNM stage, TNM 0, I and II (blue line), TNM III and IV (green line). **(C)** Overall survival curves of location, tongue (blue line), gingival and buccal mucosa (green line). **(D)** Overall survival curves of tumor size, between 0 and 2 cm (blue line), between 2 and 4 cm (green line), greater than 4 cm (gray line). **(E)** Overall survival curves of lymph node metastases, N0 (blue line), N1 (green line).** (F)** Overall survival curves of distant metastases, M0 (blue line), M1 (green line).

Univariate and multivariate analyses were performed to confirm prognostic factors for OSCC patients. Univariate analysis indicated that POM121 expression (*P*<0.001), tumor size (*P*=0.039), lymph node status (*P*<0.001), distant metastasis (*P*=0.021), and TNM stage (*P*<0.001) were significantly associated with OS of OSCC patients. Multivariate analysis showed that high POM121 expression (HR, 1.844; 95% CI, 1.321-2.575; *P*<0.001) was an independent prognostic factor for OS, as well as lymph node metastasis (HR, 1.322; 95% CI, 1.053-1.659; P=0.016) and TNM stage (HR, 1.163; 95% CI, 1.017-1.331; P=0.028) (Table 3). Kaplan-Meier survival curves confirmed that high POM121 expression, lymph node metastasis, distant metastases, and TNM stage were associated with poor OS and disease-free survival (Figure 3).

### The main enriched KEGG pathways and interacting proteins of POM121

**Figure 4:**
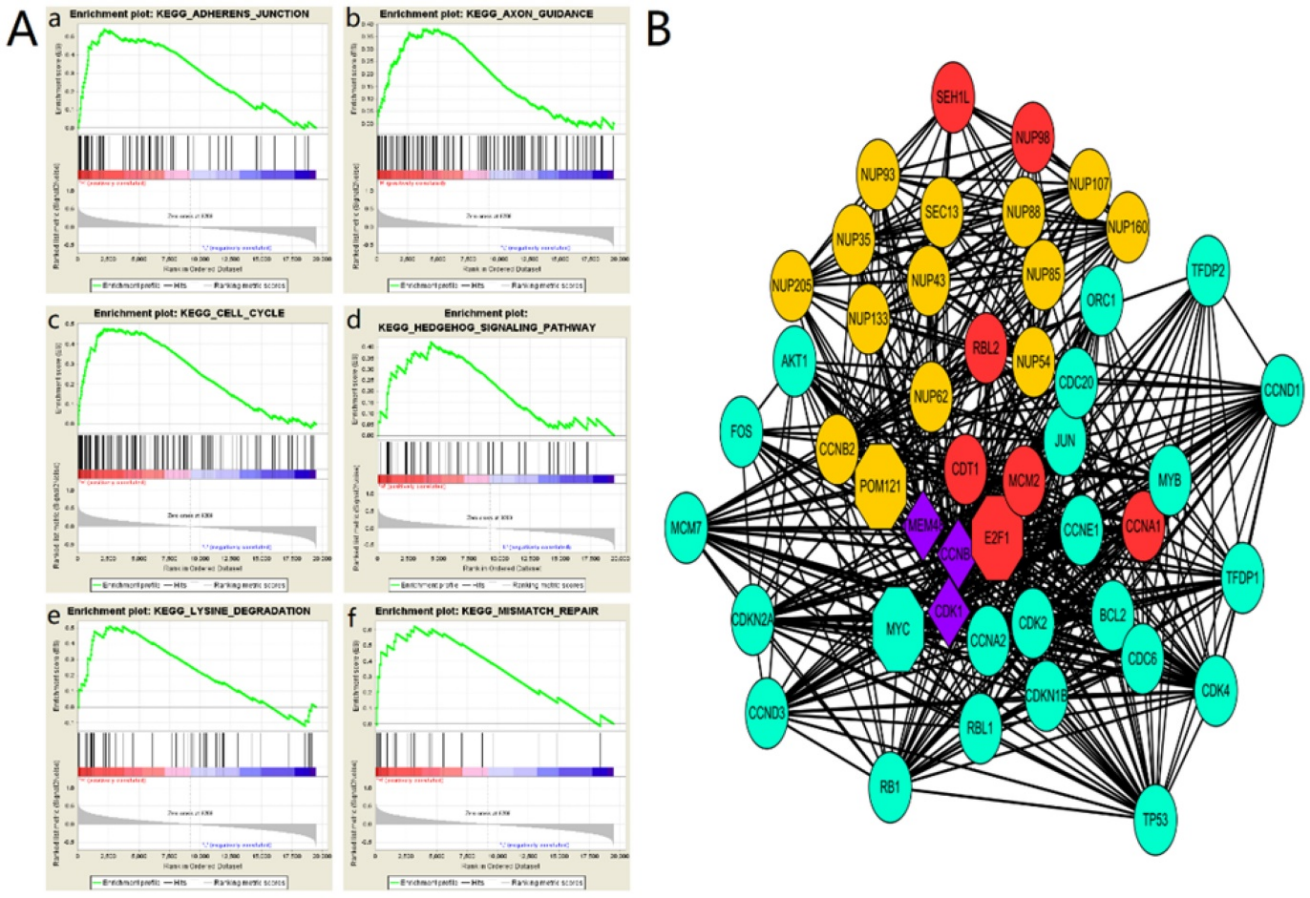
**The main enriched KEGG pathways and protein interaction network (A)**The GSEA results showing the correlation of POM121 levels and OSCC related gene sets in MSigDB. Gene sets “adherens junction”, “axon guidance”, “cell cycle”, “hedgehog signaling”, “lysine degradation” and “mismatch repair” were enriched in POM121 high expression phonotype.** (B)** Using the STRING online database, total of 47proteins (13 in Orange centring on POM121, 6 Red centring on E2F1, 22 in green centring on MYC, and 3 in purple connected with POM121, E2F1 and MYC) were filtered into the protein interaction network.

To explore the role of POM121 in OSCC proliferation and metastasis, an integrative analysis of OSCC microarray was carried out on the base of TCGA database. In the results of GSEA, high POM121 expression group is significantly enriched in adherens junction, axon guidance, cell cycle, hedgehog signaling, lysine degradation and mismatch repair (Figure 4A). We constructed a putative protein interaction network using the STRING interactive database. Each node in the network represented a protein that interacted with POM121, and different colors represent different degrees of closeness. The results showed that NUP62, NUP98, and some other proteins had interactions with POM121 (Figure 4B).

## Discussion

POM121 is a 121 kDa transmembrane NUP that anchors the NPC to the mammalian nuclear membrane and is less conserved than two other vertebrate membrane NUPs, NDC1 and GP210 20, 21. POM121 has an important role in NPC assembly, nuclear transport, and cytoplasmic membrane stacks 22-24. It was reported that the N-terminally-truncated POM121C blocks HIV-1 infection after completion of reverse transcription and before integration 25. Guo et al. further confirmed that full-length POM121 enables efficient HIV-1 pre-integration complex nuclear import by KPNB1-dependent classical cargo nuclear transportation 26. Interestingly, POM121 was also found to be involved in the propagation of nuclear apoptosis, which is related to tumorigenesis 27. POM121 was found to promote proliferation, aggressiveness, and therapeutic resistance in prostate cancer 28. However, little is known about the relationship between POM121 and oral cancer.

In this study, bioinformatic analysis revealed that POM121 and SEC13 were hub genes in OSCC tissues, with POM121 upregulation the greatest. To confirm the conclusions acquired from microarray analysis, POM121 mRNA expression was detected in fresh frozen OSCC tissues and matched non-tumorous oral tissues by qRT-PCR. The result showed that POM121 mRNA levels in OSCC tissues were significantly higher than in normal oral tissues.

According to IHC analysis, POM121 was found to be located in the nuclear envelope and nucleoplasm. High POM121 expression was detected more frequently in OSCC tissues (59.73%) than in normal oral tissues (18.75%), similar to the observed mRNA expression patterns. We also detected POM121 expression in oral dysplasia tissues and confirmed that high POM121 was expressed in OSCC tissues but not dysplasia (22.00%). Interestingly, the positive expression rates of POM121 in dysplasia did not show a significant difference with normal oral tissues. Insufficient patient samples may account for this phenomenon.

We analyzed 298 samples of OSCC tissues and their associated clinical information to explore the association between POM121 and clinicopathological features. Our findings showed that POM121 expression was significantly connected with distant metastases and TNM stage. High POM121 expression was more common in patients with distant metastases and TNM (III + IV) stage. These results indicated that POM121 was associated with the development and progression of OSCC. Moreover, the correlation between POM121 protein expression and distant metastasis indicated POM121 may be involved in OSCC metastasis.

Univariate Cox regression analysis showed that POM121 expression, tumor size, lymph node status, distant metastasis, and TNM stage were associated with survival of OSCC patients. Multivariate analysis further confirmed that POM121 expression, lymph node metastasis, and TNM stage were independent prognostic factors for OSCC patients. Kaplan-Meier survival curves also supported the importance of POM121 in patient prognosis. These results suggested that high levels of POM121 in patients predicted poor OS and disease-free survival.

Little is known about the molecular mechanisms of POM121 in OSCC development and progression. The results of GSEA showed that the expression of POM121 influences adherens junction, axon guidance, cell cycle, hedgehog signaling, mismatch repair and lysine degradation. Mismatch repair is well-known biological activity involved in cancer, and the role of mismatch repair and relevant proteins in oral carcinogenesis and development has grown 29. Adherens junction refers to the feature of all epithelial sheets and apical adhesive structures, regulating cancer invasion and metastasis 30. Hedgehog signaling is reported to modulate the tumor microenvironment in several cancers, associated with tumor cell growth, invasion, and metastasis 31, 32. Hence, we presumed POM121 may participate in tumorigenesis, invasion and metastasis.

Our protein interaction network revealed close connection between POM121 and a large group of proteins, including NUP62, MYC and so on. POM121 may participate in tumor progression through these proteins. The protein network also showed POM121 interacted with NUP62. Interestingly, NUP62 is confirmed to regulate squamous cell carcinoma proliferation by TP63 (a p53 homolog)^33^. POM121 regulates nuclear import of oncogenic MYC through importinβ to promote prostate cancer 28. Some studies demonstrated MYC is involved in tumorigenesis and progression in OSCC 34-36. Furthermore, it has been confirmed that soluble POM121 has a role in NUP98-mediated gene regulation by colocalizing and interacting with the transcriptional regulator NUP98, which has been found to participate in the pathogenesis of several hematological malignancies 37, 38. NUP98 is required for full expression of p21, a critical effector of the p53 pathway, which is associated with OSCC progression 39, 40. Further studies should to be undertaken to explore if POM121 regulates the expression of MYC and/or p53 signaling pathways.

## Conclusion

The expression of POM121 in OSCC tissues is higher than that in normal oral tissues. High POM121 expression is associated with poor overall survival in OSCC patients. Our results show that POM121 has the potential to be considered as an independent prognostic biomarker in OSCC.

## Figures and Tables

**Table 1:** POM121 expression in oral tissues

Characteristic	n	POM121 expression(%)		χ^2^	*P*
		Low or no	High		
				39.233	<0.001*
Oral cancers	298	120(40.27)	178(59.73)		
Normal	32	26(81.25)	6(18.75)		
Dysplasia	50	39(78.00)	11(22.00)		

**Table 2:** Association of POM121expression with clinicopathological characteristics in OSCC patients

Characteristics	n	POM121 expression (%)		Pearson χ2	*P* value
		Low or no (%)	High (%)		
Total	298	120(40.27)	178(59.73)		
Gender				2.731	0.098
male	134	47(25.07)	87(64.93)		
female	164	73(44.51)	91(55.49)		
Age				1.417	0.234
≥ 60	159	59(37.11)	100(62.89)		
<60	139	61(43.88)	78(56.12)		
Localization				0.214	0.643
tongue	179	74(41.34)	105(58.66)		
other^a^	119	46(38.66)	73(61.34)		
Differentiation				3.109	0.211
well	216	92(42.59)	124(57.41)		
moderate	71	26(36.62)	45(63.38)		
poor	11	2(18.12)	9(81.82)		
Tumor size				0.339	0.844
T1≤2cm	178	71(39.89)	107(60.11)		
2cm<T2≤4cm	108	45(41.67)	63(58.33)		
T3>4cm	12	4(33.33)	8(66.67)		
Lymph node metastases				4.924	0.085
N0	245	101(41.22)	144(58.78)		
N1	18	10(55.56)	8(44.44)		
N2	35	9(25.71)	26(74.29)		
Distant metastases				7.680	0.006*
M0	275	117(42.55)	158(57.45)		
M1	23	3(13.04)	20(86.96)		
TNM stage				5.352	0.021*
0+I+II	185	84(45.41)	101(54.59)		
III+IV	113	36(31.86)	77(68.14)		
Caries				0.622	0.430
Yes	58	26(44.83)	32(55.17)		
No	240	94(39.17)	146(60.83)		
Smoking				0.410	0.522
Yes	26	12(41.15)	14(53.85)		
No	272	108(39.71)	164(60.29)		

a, Areas other than the tongue, such as gingiva, buccal mucosa, floor of mouth, lip, etc.

**Table 3:** Univariate and multivariate analysis of prognostic factors for overall survival in oral cancer

	Univariate analysis			Multivariate analysis		
	HR	*P* -value	95%CI	HR	*P*-value	95%CI
POM121 expression						
high vs low or no	1.903	0.000*	1.384 2.617	1.844	0.000*	1.321 2.575
Gender	1.124	0.433	0.839 1.505			
male vs female						
Age(year)	1.119	0.449	0.836 1.498			
≤60 vs> 60						
Location	1.106	0.500	0.825 1.485			
Tongue vs Others						
Differentiation	1.063	0.637	0.824 1.372			
well vs moderate vs poor						
Tumor size	1.289	0.039*	1.013 1.640			
T1vs T2 vs T3 vs T4^b^						
Lymph node metastasis	1.493	0.000*	1.223 1.823	1.322	0.016*	1.053 1.659
N0 vs N1 vs N2 vs N3						
Distant metastasis	1.775	0.021*	1.090 2.891			
M0 vs M1						
TNM stage	1.285	0.000*	1.160 1.424	1.163	0.028*	1.017 1.331
I + II vs III+ IV						
Caries	0.690	0.075	0.458 1.038			
yes vs no						
Smoking	0.571	0.071	0.310 1.048			
yes vs no						

HR, hazard ratio, CI, confidence interval. T4^b^, invasion of adjacent area.

**Table 4:** The main enriched KEGG pathways of POM121

Term	ES	NES	*P*-value	FDR q-value
KEGG_ADHERENS_JUNCTION	0.54	1.83	0.006	0.268
KEGG_AXON_GUIDANCE	0.38	1.51	0.037	0.365
KEGG_CELL_CYCLE	0.48	1.66	0.040	0.298
KEGG_HEDGEHOG_SIGNALING_PATHWAY	0.42	1.51	0.049	0.329
KEGG_LYSINE_DEGRADATION	0.51	1.69	0.014	0.318
KEGG_MISMATCH_REPAIR	0.62	1.63	0.044	0.297

ES: enrichment score; NES: normalized enrichment score; FDR: false discovery rate.
